# Preparation of phosphines through C–P bond formation

**DOI:** 10.3762/bjoc.10.106

**Published:** 2014-05-09

**Authors:** Iris Wauters, Wouter Debrouwer, Christian V Stevens

**Affiliations:** 1 Research Group SynBioC, Department of Sustainable Organic Chemistry and Technology, Faculty of Bioscience Engineering, Ghent University, Coupure links 653, 9000 Ghent, Belgium

**Keywords:** cross-coupling, enantioselectivity, hydrophosphination, organophosphorus chemistry, phosphines, phosphine-boranes, substitution reactions, trivalent phosphorus

## Abstract

Phosphines are an important class of ligands in the field of metal-catalysis. This has spurred the development of new routes toward functionalized phosphines. Some of the most important C–P bond formation strategies were reviewed and organized according to the hybridization of carbon in the newly formed C–P bond.

## Introduction

Phosphines are an important class of organophosphorus compounds. They are often used as ligands in metal complex catalysis and they have become a popular reagent for organocatalysis [1]. The methods most widely used for the synthesis of phosphines include the reaction of organometallic compounds with halophosphines, the reaction of metal phosphides with alkyl halides, the reduction of other phosphorus compounds and the hydrophosphination [2]. Research in the past years has focused on the catalytic synthesis of phosphines [3–4]. The asymmetric catalytic synthesis of chiral phosphines has only recently emerged and is under full development. Chiral phosphines are interesting ligands for the preparation of transition metal complex catalysts for asymmetric synthesis [5–6]. Only a minor part of the chiral phosphines are chiral at the phosphorus atom (*P*-stereogenic) [7–9].

A major drawback of phosphines is their highly oxidizable nature. They are easily converted to the corresponding phosphine oxide which makes the isolation difficult. To prevent losses during purification, the phosphines are sometimes deliberately transformed into the corresponding oxides (or sulfides). However, this requires an additional reduction step afterwards to get the phosphine back [10–15]. Therefore phosphines are sometimes protected by generation of the corresponding phosphine–borane complex [16–17]. The phosphine–borane complex is a stable intermediate toward the free phosphine. If necessary the boranato group can be removed by treatment with an excess of amine [18]. However, not all phosphines are prone to oxidation and show good air-stability [19].

This review will provide a general overview on phosphine synthesis over the last 10 to 15 years. Only reactions establishing a C–P bond will be discussed. The synthesis of phosphine-based polymers was not included [20]. Reactions involving pentavalent phosphorus derivatives (phosphine oxides, phosphonates, phosphinates and phosphate derivatives, etc.) are out of the scope of this review.

## Review

### Preparation of alkylphosphines via formation of a C(sp^3^)–P bond

#### Reaction of organometallic reagents with halophosphines

One of the main approaches to synthesize a carbon–phosphorus bond involves the displacement of a halogen atom from phosphorus by an organometallic reagent. This method has proven its usefulness for many years. A variety of organometallic compounds have been described. Most frequently used are the Grignard [21–22] and lithium species. But also organozinc [23–24], organolead [25], organomercury [26] or aluminum-based [27] reagents have been used. However, nowadays it is recommended to avoid the use of certain reagents such as organomercury or organolead compounds as they pose a serious toxicological hazard [28–29].

Despite the fact that the methodology is historically useful it also has major drawbacks. The presence of an anionic carbon reagent in the reaction restricts the scope of the methodology. The aspired phosphines cannot contain certain functional groups that are able to react with the organometallic compound. Further, stoichiometric amounts of reagents are required. Also, attention should be paid to the handling of halophosphines as some of the simple alkyldichlorophosphines are extremely corrosive and flammable in air.

Asymmetric phosphines are difficult to access via a nucleophilic substitution at a halophosphine due to the limited availability of unsymmetrical halophosphines and their weak configurational stability. *P*-stereogenic chlorophosphines racemize easily even at room temperature [30].

Enantiopure *P-*stereogenic compounds can be synthesized via a diastereoselective nucleophilic substitution at phosphorus utilizing chiral auxiliaries. Diastereomeric intermediates are formed that are separable by chromatography or recrystallization. The protocol has proven to be effective and has become the preferred approach for the synthesis of chiral phosphines. Commonly used chiral auxiliaries are chiral secondary alcohols (for example (−)-menthol (**3**), *endo*-borneol, etc.) or thiols that are reacted with halophosphines [31–34].

**Scheme 1:**
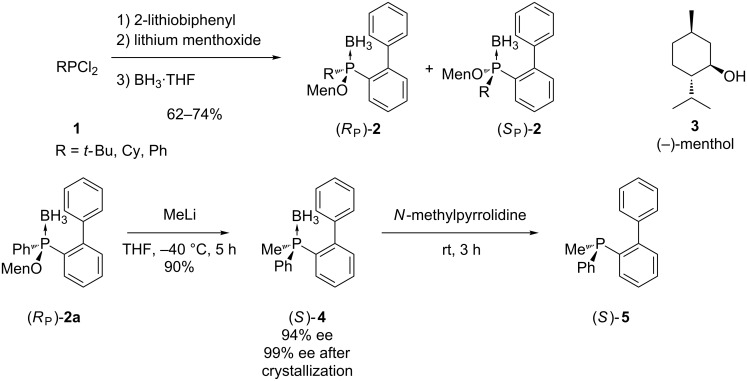
Synthesis of *P-*stereogenic phosphines **5** using menthylphosphinite borane diastereomers **2**.

The diastereoisomers of menthylphosphinite boranes are popular synthetic intermediates for this approach (Scheme 1) [35]. The diastereomeric phosphinites **2**, that were prepared from an alkyldichlorophosphine **1**, were separated by preparative HPLC or recrystallization. Nucleophilic substitution of pure diastereomer (*R*_P_)-**2a** with methyllithium afforded the phosphine–borane (*S*)-**4** with 94% enantiomeric excess. The substitution resulted in inversion of the configuration at the phosphorus center. Deboranation of the air stable borane adduct (*S*)-**4** to obtain **5**, was achieved by treatment with *N*-methylpyrrolidine.

**Scheme 2:**
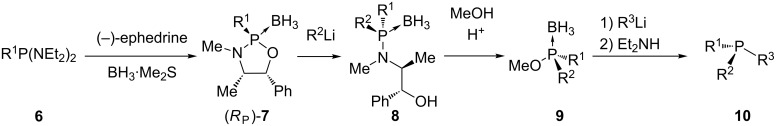
Enantioselective synthesis of chiral phosphines **10** with ephedrine as a chiral auxiliary.

An alternative method is based on ephedrine as a chiral auxiliary and was developed by Genêt and Jugé [36–37]. The key synthetic intermediates in this approach are 1,3,2-oxazaphospholidine boranes **7**. These compounds are the result of the reaction between bis(diethylamino)alkylphosphine **6** and ephedrine, followed by protection with borane. The subsequent stereoselective ring opening of compound **7** with an organolithium reagent gives way to acyclic products **8** with retention of configuration at the phosphorus center. These phosphamide boranes **8** undergo methanolysis with inversion of configuration to produce intermediate phosphinite boranes **9** that are subsequently substituted with a second nucleophile. A following deprotection of the boranato group gives the chiral phosphines **10**. Both enantiomers can be obtained by preparation of different starting oxazaphospholidine borane complexes **7** from (*−*)-ephedrine or (*+*)-ephedrine [38] or by starting from the same oxazaphospholidine borane adduct **7** and then changing the order of addition of the organolithium reagents (Scheme 2).

**Scheme 3:**
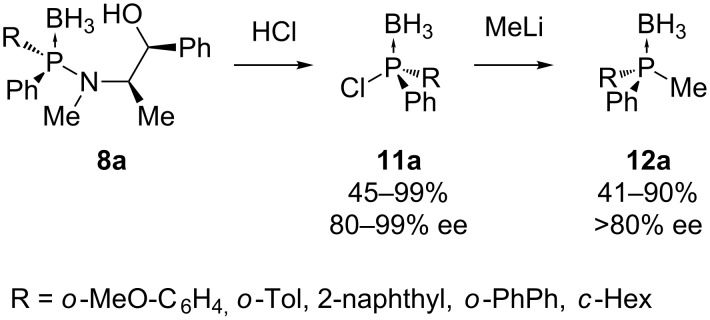
Chlorophosphine boranes **11a** as *P-*chirogenic electrophilic building blocks.

Acidolysis with HCl of compounds **8a** results in the stereoselective synthesis of chiral chlorophosphine boranes **11a** [39]. The borane complex has a good configurational stability with borane as a protecting group, in contrast to chlorophosphines that can undergo inversion at the phosphorus center [30]. They allow the synthesis of a variety of *P-*chiral tertiary phosphine boranes **12a** via substitution of the chlorine atom with organometallic nucleophiles. This substitution causes an inversion of configuration at the phosphorus center (Scheme 3). Schuman et al. have prepared several dialkenylphosphines using this methodology [40].

#### Nucleophilic substitution with metallated organophosphines

Another classical method for the preparation of phosphines is the nucleophilic substitution of alkyl halides with phosphide anions derived from secondary phosphines or phosphine–borane complexes [41]. This approach requires stoichiometric amounts of base. Numerous examples of this approach are available [22,42–48].

In recent years methodologies were developed for the asymmetric alkylation. Livinghouse and Wolfe have reported an enantioselective method for the preparation of chiral tertiary phosphine–boranes starting from a racemic secondary phosphine borane precursor such as **13a** (Table 1) [49]. A nucleophilic phosphide reagent was prepared by deprotonation of **13a** in the presence of (*−*)-sparteine. The subsequent alkylation of the lithium phosphide with an electrophile proceeded with good enantiocontrol via dynamic resolution. One enantiomer is thermodynamically favored by the spartein auxiliary. The enantioselectivity was found to be time and temperature dependent. Simple stirring of the intermediate (*−*)-sparteine–lithium complex of **13a** for 1 h at 25 °C prior to alkylation resulted in an increase in enantiomeric excess of **14a**.

**Table 1:** Alkylations of dynamically resolved *tert*-butylphenylphosphine borane **13a**.

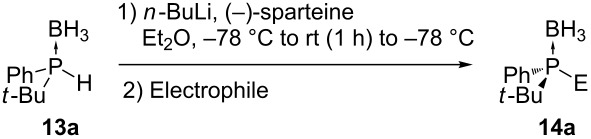			

Entry	Electrophile	Yields of **14a** (%)	ee of **14a** (%)

1	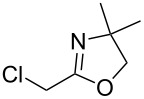	90	>82
2	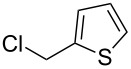	85	95
3	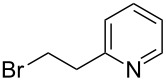	90	92

**Scheme 4:**
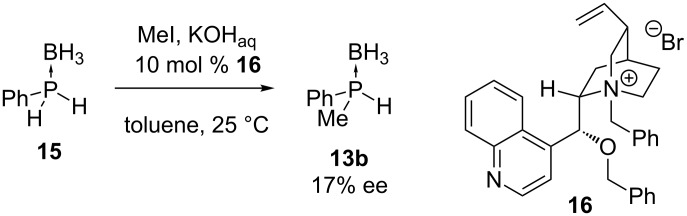
Monoalkylation of phenylphosphine borane **15** with methyl iodide in the presence of *Cinchona* alkaloid-derived catalyst **16**.

The organocatalyst **16** has also been used to carry out an asymmetric alkylation reaction (Scheme 4). The monoalkylation of phosphine–borane complex **15** was performed in the presence of the *Cinchona* alkaloid ammonium salt **16** [50]. However, the enantioselectivity of the reaction was low.

**Scheme 5:**
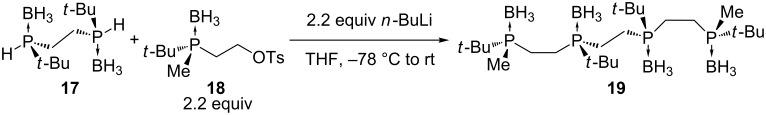
Preparation of tetraphosphine borane **19**.

Imamoto et al. prepared a new tetraphosphine ligand **19** by deprotonation of enantiopure secondary diphosphine borane **17** at low temperature (Scheme 5) [51]. The configuration was retained during the nucleophilic attack at **18**. This approach provides a very straightforward access to *P*-stereogenic tertiary phosphines but requires the availability of *P*-chiral substrates.

**Scheme 6:**
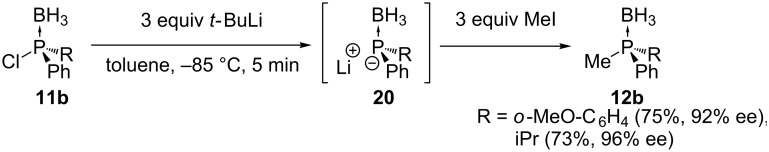
Using chiral chlorophosphine-boranes **11b** as phosphide borane **20** precursors.

Jugé and co-workers synthesized chiral tertiary phosphine–borane complexes **12b** starting from *P-*stereogenic chlorophosphine–borane complexes **11b** (Scheme 6) [52]. These complexes are accessible with the ephedrine methodology (vide supra). Treatment of **11b** with *t*-butyllithium leads to metal–halogen exchange. After reaction of the phosphide anion **20** with an electrophile, the chiral tertiary phosphine boranes **12b** are formed with retention of configuration at the phosphorus atom.

#### Catalytic C(sp^3^)–P bond formation

Only a few examples of a metal catalyzed C(sp^3^)–P cross-coupling exist and they are mostly restricted to benzylic and allylic coupling partners.

**Scheme 7:**
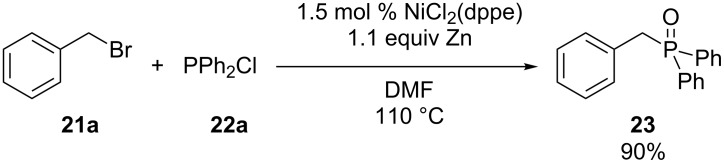
Nickel-catalyzed cross-coupling (dppe = 1,2-bis(diphenylphosphino)ethane).

Ager and Laneman have synthesized tertiary phosphine oxide **23** through the nickel-catalyzed coupling of benzyl bromide (**21a**) with diphenylphosphine chloride (**22a**) (Scheme 7) [53]. However oxidation occurred during work-up.

The group of Togni has investigated a palladium-catalyzed enantioselective coupling reaction between allylic substrates **24** and several secondary phosphines **25a** as nucleophiles [54]. The scope of the reaction was limited to 1,3-diphenylallyl acetate **24**. The reaction produced not only **26**, but gave several side products **27**–**29** (Table 2).

**Table 2:** Palladium-catalyzed asymmetric allylic phosphination (dba = dibenzylideneacetone).

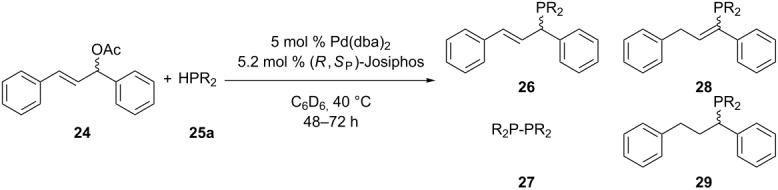				

Entry	R	**26**:**27**:**28**:**29** (%)	Yield of **26** (%)	ee of **26**(%)

1	Ph	89:11:0:0	79	96
2	Cy	65:28:6:1	44	45
3	2-naphthyl	91:6:1:2	85	83
4	*o*-Tol	88:8:2:2	82	42

**Scheme 8:**
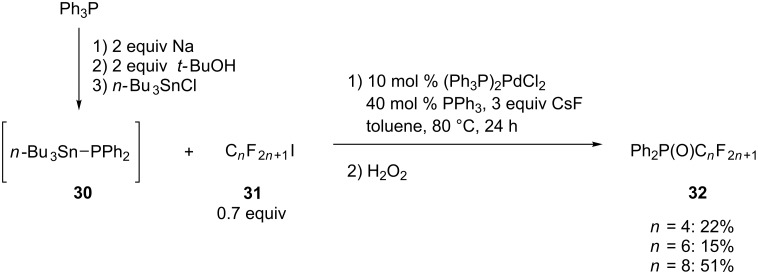
Pd-catalyzed cross-coupling reaction with organophosphorus stannanes **30**.

Another example of a C(sp^3^)–P cross-coupling was reported by Lanteri et al. [55]. A palladium catalyst effectuated the coupling of *n*-Bu_3_SnPPh_2_ (**30**) with several perfluoroalkyl iodides **31** (Scheme 8). The stannane **30** was in situ generated by the reaction of the diphenylphosphide anion with *n*-Bu_3_SnCl. After oxidation, the perfluoroalkyl-substituted phosphine oxides **32** were obtained in low to moderate yields (15–51%) although full conversion was observed. The byproduct formed was reduced perfluoroalkane HC*_n_*F_2_*_n_*_+1_.

**Scheme 9:**
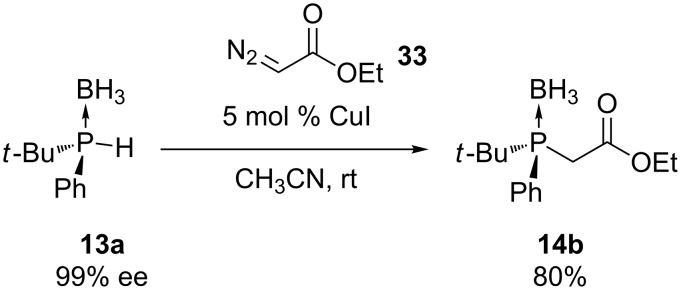
Copper iodide catalyzed carbon–phosphorus bond formation.

Ethyl diazoacetate (**33**) was reacted with the secondary phosphine borane **13a** in the presence of a copper catalyst [56]. The product **14b** was obtained in good yield with retention of configuration at the phosphorus center (Scheme 9). Other chiral phosphine boranes **13** were reacted similarly. This protocol is limited to the availability of these chiral substrates.

**Scheme 10:**
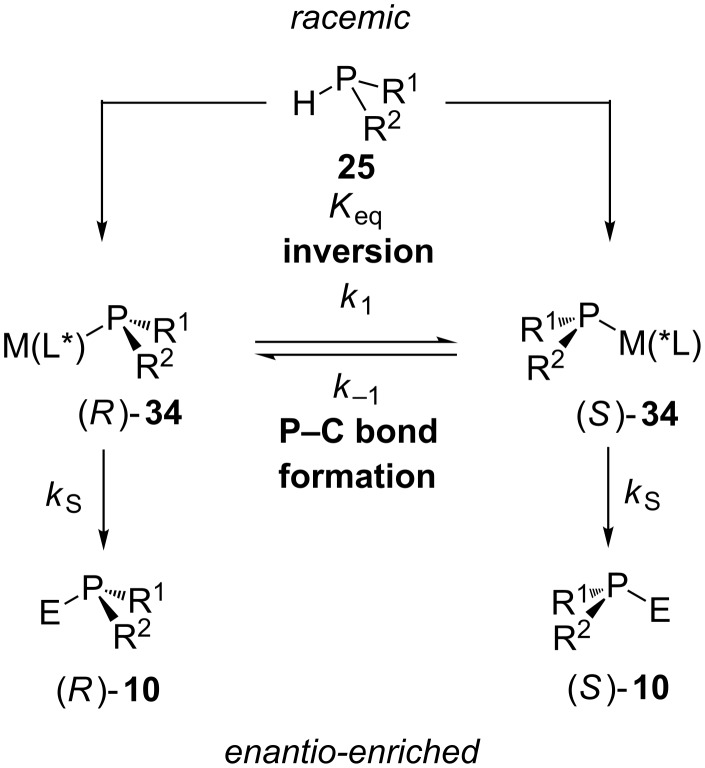
Thermodynamic kinetic resolution as the origin of enantioselectivity in metal-catalyzed asymmetric synthesis of *P-*stereogenic phosphines.

Protocols for the enantioselective cross-coupling of benzyl or alkyl halides with racemic secondary phosphines have been developed. These reactions were catalyzed by chiral platinum or ruthenium complexes. The enantioselectivity is based on a dynamic kinetic resolution. Upon reaction with the catalyst precursor containing a chiral ligand (L*), a diastereomeric metal–phosphido complex **34** is formed. Rapid pyramidal inversion of this key catalytic intermediate **34** occurs. This complex performs a nucleophilic attack on the electrophile resulting in tertiary phosphines **10**, in which the substituent ‘E’ comes from the electrophile. If the inversion of the diastereomers **34** is much faster than their reactions with an electrophile, *P-*stereogenic phosphines **10** are formed enantioselectively. The ratio of phosphine end products **10** is determined by the equilibrium (*K*_eq_) between the complexes **34** and the rate of nucleophilic attack (*k*_S_ and *k*_R_) on the electrophile. The enantioselectivity of the end products **10** is related to the ratio of the diastereomeric phosphido complexes **34**. The major phosphine product is derived from the major diastereomeric phosphido complex. The dynamic kinetic resolution approach has been reviewed in more detail by Glueck [57–58]. Scheme 10 relates to reactions of secondary phosphines with several electrophiles, including alkyl halides (alkylation), alkenes (hydrophosphination) and aryl iodides (arylation).

**Scheme 11:**
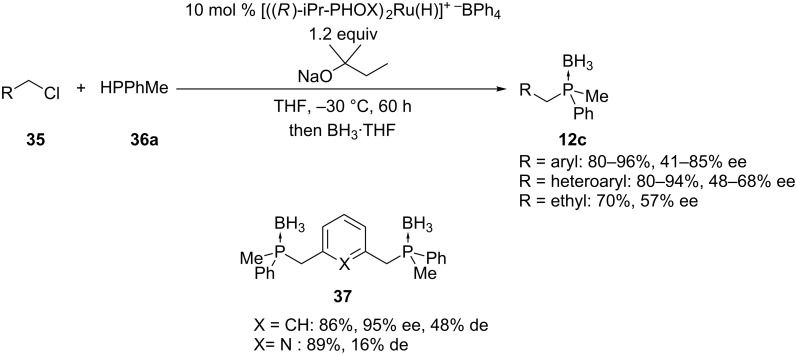
Ru-catalyzed asymmetric phosphination of benzyl and alkyl chlorides **35** with HPPhMe (**36a**, PHOX = phosphinooxazoline).

Chan et al. synthesized *P-*stereogenic phosphine boranes using a ruthenium catalyst. The secondary phosphine **36a** underwent an enantioselective alkylation to **12c** (Scheme 11). The mechanism of the reaction is based on the formation of an electron-rich ruthenium–phosphido complex that enhances the nucleophilicity at the phosphorus atom. This permitted the reaction to proceed with the less electrophilic benzylic chlorides **35** instead of bromides. The metal-catalyzed reaction was faster than the achiral base-mediated alkylation of **36a**. Bisphosphines **37** were also reported with high enantiomeric excesses. The procedure is mainly restricted to benzylic halides but also allowed for the asymmetric alkylation with ethyl bromide. All the phosphines were isolated as their air-stable phosphine–borane complexes **12c**, **37** [59–60].

**Scheme 12:**
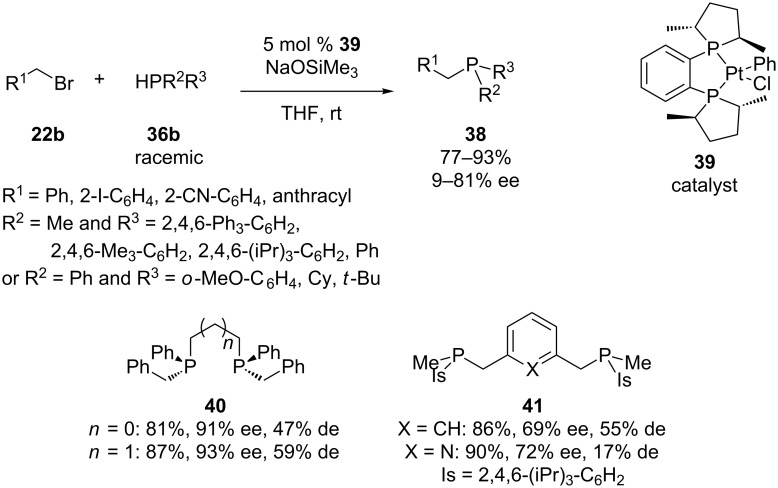
Pt-catalyzed asymmetric alkylation of secondary phosphines **36b**.

The group of Glueck has reported a method for the asymmetric alkylation of racemic secondary phosphines **36b** by means of a chiral platinum-based catalyst **39** (Scheme 12) [61]. The enhanced nucleophilicity at phosphorus of the platinum–phosphido intermediate was beneficial for the alkylation. The scope of the reaction was investigated using diverse benzylic bromides **22b** and secondary phosphines **36b**. Bidentate ligands **40** and **41** were also synthesized [61–62]. This procedure was also restricted to benzylic halides. High enantiomeric excesses were reported. As expected, a mechanistic study suggested that the major enantiomer of product was formed from the major diastereomer of the platinum–phosphido intermediate [63]. Glueck and co-workers also developed an analogous method for the tandem alkylation/arylation of primary phosphines on the basis of a platinum catalyst resulting in several enantio-enriched phosphaacenaphtalenes [64].

#### Hydrophosphination

Hydrophosphination involves the addition of P–H to an unsaturated C–C bond. In this reaction phosphines, silylphosphines [65–66] or phosphine–borane complexes are used as phosphinating agents to react with unactivated or activated alkenes, dienes and alkynes. Hydrophosphination has gained much interest as an alternative to the classical phosphine syntheses involving a substitution that is incompatible with certain functional groups. Moreover the addition of P–H to an unsaturated C–C bond is more efficient than substitution reactions when considering atom efficiency, what makes it not only greener but also more economical. Other phosphination reactions of unsaturated bonds, such as diphosphination, thiophosphination or selenophosphination, were not included [67].

**Scheme 13:**
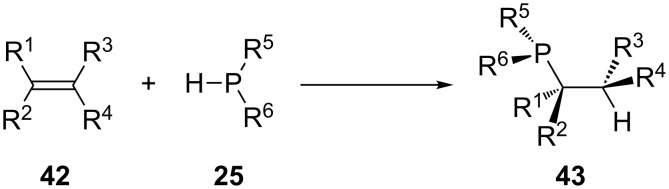
Different adducts **43** can result from hydrophosphination.

Depending on the regioselectivity of the reaction, the addition of P–H to the unsaturated bond results in the formation of different products **43** (Scheme 13). The product that results from the Markovnikov addition of P–H corresponds to the α-adduct and the anti-Markovnikov addition is referred to as the β-adduct. The stereoselectivity of the method determines the conformation at the newly formed chiral centers.

The hydrophosphination typically proceeds via thermal [68–69], radical, acidic [70–72] or basic [73–74] initiation. Radical addition of secondary phosphines to alkenes can be accomplished by thermal activation [75–76], through the use of radical initiators (AIBN) [77–82] or photochemically by irradiation with UV or visible light [22,83–85]. Most of these reactions give anti-Markovnikov products. The hydrophosphination of activated alkenes (e.g., Michael acceptors) has also been shown to take place at room temperature in the absence of a catalyst [86–87] and even under solvent-free conditions [88]. More recently also metal complex-assisted or organocatalyzed hydrophosphinations have been reported. Several reviews focusing on hydrophosphination have been pusblished [89–91].

In recent years a lot of progress has been made in the metal complex-catalyzed hydrophosphination. It was shown that several metals can function as catalysts for the inter- and intramolecular addition of PH_3_ and R_2_PH to alkenes. Most research has focused on the use of platinum [92–96], palladium [97–99] or nickel [100–104] complexes. Other catalysts that have been less investigated are iron [105–107], rhodium [108–110], lanthanides [111–114], copper [115] and alkaline-earth metals [114,116]. The catalyst activates either the P*-*nucleophile or the C-electrophile.

Chiral phosphines have attracted more and more interest since they are employed as ligands in transition metal complexes to perform asymmetric catalysis [117]. Enantiopure phosphines have mostly been prepared by starting from enantiopure products or by resolution. The methodologies for catalytic asymmetric hydrophosphination of olefins are limited. Chiral metal complexes have been used to promote and control the asymmetric P–H addition reaction. Recent reviews covering the asymmetric hydrophosphination reaction catalyzed by metal catalysts have been published by Glueck [118–119] and Pullarkat and Leung [120]. Some recent developments in the asymmetric catalytic hydrophosphination will be discussed.

**Scheme 14:**
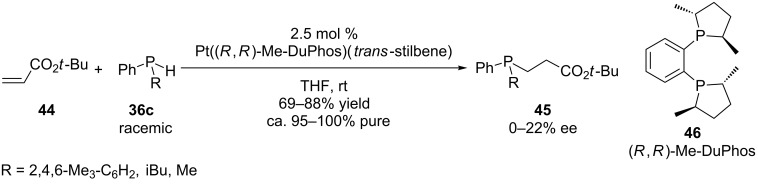
Pt-catalyzed asymmetric hydrophosphination.

The group of Glueck reported on an approach to chiral phosphines by the addition of secondary phosphines **36c** to Michael acceptor alkenes (acrylonitrile or derivatives and acrylate esters **44**) in the presence of Pt((*R*,*R*)-Me-DuPhos) complexes (Scheme 14). However, the products **45** suffered from low enantioselectivities [121]. The mode of action is based on the activation of the P*-*nucleophile. The proposed mechanism includes the P–H oxidative addition to platinum giving a platinum–phosphido complex. Subsequent nucleophilic attack on a Michael acceptor alkene was suggested to lead to a zwitterion intermediate. Addition of a protic additive was beneficial for the selectivity and reaction rate [95].

**Scheme 15:**
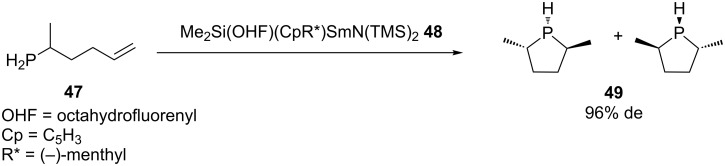
Intramolecular hydrophosphination of phosphinoalkene **47**.

Several chiral cyclic phosphines were acquired via the lanthanide catalyzed intramolecular hydrophosphination of phosphinoalkenes. Scheme 15 shows the diastereoselective synthesis of 2,5-dimethylphospholanes **49** from **47** with a lanthanide catalyst **48** [122]. The common mechanism when using lanthanide [113] or alkaline earth metal [123] catalysts is based on the formation of a phosphido–metal complex that undergoes insertion of the olefin. Protonolysis of the metal–alkyl complex via σ-bond metathesis with the phosphine reagent completes the catalytic cycle giving the product and regenerating the phosphido intermediate.

The group of Togni has developed an enantioenriched hydrophosphination of vinyl nitriles catalyzed by a dicationic nickel complex (Table 3). The method is based on the activation of the electrophile. It was suggested that complexation of the nitrile **50** to the chiral nickel Lewis acid activates the double bond towards 1,4-addition of the phosphine **25b**. A final proton transfer yields the phosphine product **51** [124–125].

**Table 3:** Ni-catalyzed asymmetric hydrophosphination of methacrylonitrile **50**.

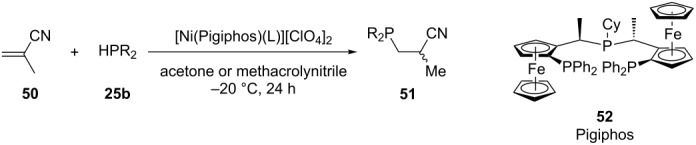			

Entry	R	Yield of **51** (%)	ee of **51** (%)

1	Ph	10	32
2	Cy	71	70
3	iPr	not isolated	78
4	Ad	95	94
5	*t-*Bu	97	89
6	EtMe_2_C-	86	90

A chiral Pincer-palladium complex **55** has been used for the addition of diarylphosphines **25c** to enones **53** (Table 4) [126]. Several enones **53**, having electron-donating or -withdrawing groups on the aromatic ring, reacted with a variety of electron-rich and -poor diarylphosphines **25c**. The chiral phosphine oxides **54** were obtained in high yield with excellent stereoselectivities. In the proposed mechanism the catalyst **55** acts as a base toward the diarylphosphine **25c**. Some other examples of palladium-catalyzed asymmetric hydrophosphination are the addition of diphenylphosphine to α,β-unsaturated ketones [127–128], esters [129], sulfonic esters [130] or to dienones [131]. The proposed mechanism is ubiquitous in metal-catalyzed hydrophosphination involving a P–H oxidative addition, insertion of the olefin into the Pd–H bond and reductive elimination.

**Table 4:** Palladium-catalyzed asymmetric addition of diarylphosphines **25c** to enones **53**.

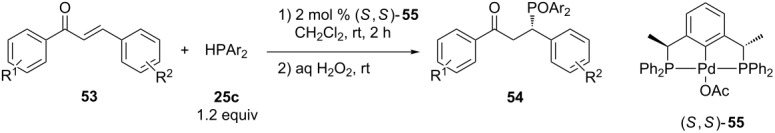					

Entry	R^1^	R^2^	Ar	Yield of **54** (%)	ee of **54** (%)

1	H	H	Ph	93	99
2	*p*-Br-	H	Ph	89	99
3	*p*-MeO-	H	Ph	75	98
4	*m*-Br-	H	Ph	93	97
5	*p*-NO_2_-	H	Ph	78	95
6	H	*p*-Br-	Ph	90	98
7	H	*p*-NO_2_-	Ph	88	99
8	H	*m*-Br-	Ph	90	99
9	H	*o*-MeO-	Ph	69	90
10	H	*p*-Me-	Ph	63	90
11	H	H	*p*-MeO-C_6_H_4_	86	94
12	H	H	*p*-Cl-C_6_H_4_	92	96

In 2007 several papers appeared reporting on organocatalyzed asymmetric hydrophosphinations. The organocatalytic process has the advantage that in contrast to a metal-catalyzed method, it cannot undergo product inhibition as a result of the coordination ability of phosphorus to a metal catalyst.

The addition of diphenylphosphine to a range of nitroalkenes **56** has been described using a bifuntional *Cinchona* alkoid/thiourea catalyst **58** [132]. The catalyst **58** is able to simultaneously activate both the electrophilic and nucleophilic reagents. On one hand the thiourea presumably binds the nitro group while on the other hand the tertiary amine enables proton transfer from phosphorus to carbon (Table 5).

**Table 5:** Organocatalytic asymmetric hydrophosphination of nitroalkenes **56**.

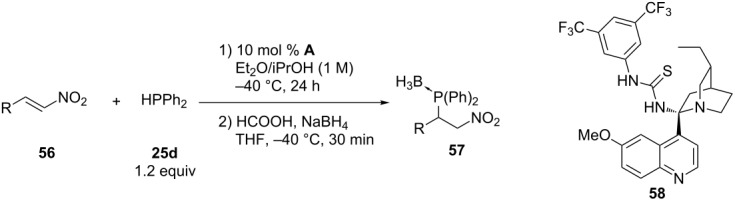					

				After crystallizaton	
Entry	R	Yield of **57** (%)	ee of **57** (%)	Yield of **57** (%)	ee of **57** (%)

1	Ph-	86	67	36	99
2	*p*-Me-C_6_H_4_-	67	52		
3	*o*-F-C_6_H_4_-	83	45	24	99
4	*o*-BnO-C_6_H_4_-	90	60	37	99

**Scheme 16:**
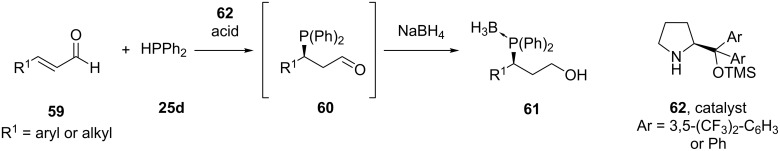
Organocatalytic asymmetric hydrophosphination of α,β-unsaturated aldehydes **59**.

The organocatalyzed hydrophosphination of α,β-unsaturated aldehydes has been described by Carlone et al. [133] and Ibrahem et al. [134]. The method is based on activation of the aldehyde **59** via iminium-ion formation by reaction with chiral pyrrolidine **62** derivatives and acid (Scheme 16). Subsequent treatment with sodium borohydride forms the air-stable phosphine–borane product and also reduces the aldehyde. The method gives compounds **61** in high yields and enantioselectivities (ee up to 99%) for α,β-unsaturated aldehydes containing either aliphatic or aromatic groups.

### Preparation of alkenylphosphines via formation of a C(sp^2^)–P bond

The C(sp^2^)–P bond formation is reviewed for arylic and vinylic phosphines. The group of Gaumont has provided a recent review (2010) on the main synthetic methods to obtain alkenylphosphines [135].

#### Reaction of organometallic reagents with halophosphines

The reaction of an organometallic reagent with the P*-*atom of halophosphines is a classical method used for the synthesis of both alkenyl- and arylphosphines. The organometallic reagents are mostly Grignard reagents [136–138] or organolithium [139–142] derivatives. Other organometallic reagents such as aluminum [143] or organomercury [26,144] reagents have been used less frequently.

Grignard or organolithium compounds are highly reactive nucleophiles and do not tolerate the presence of various functional groups. As a consequence, new approaches were developed including zinc, zirconium and copper reagents.

**Scheme 17:**
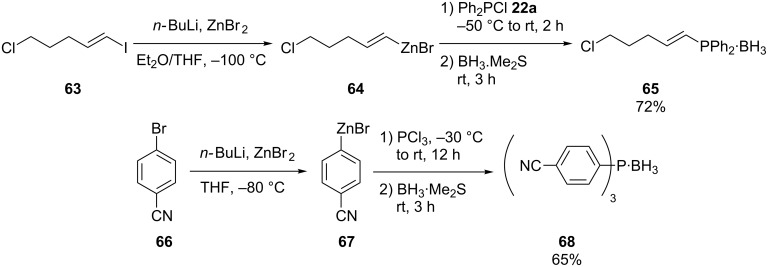
Preparation of phosphines using zinc organometallics.

Polyfunctional alkenylphosphine **65** was accessible via the reaction of organozinc derivative **64** with chlorophosphine **22a**. The organozinc bromide **64** was prepared from the corresponding alkenyl iodide **63**. To prevent oxidation, the phosphines were protected as the corresponding borane adducts **65**. The methodology is also applicable for aryl bromide **66** (Scheme 17) [23–24].

**Scheme 18:**
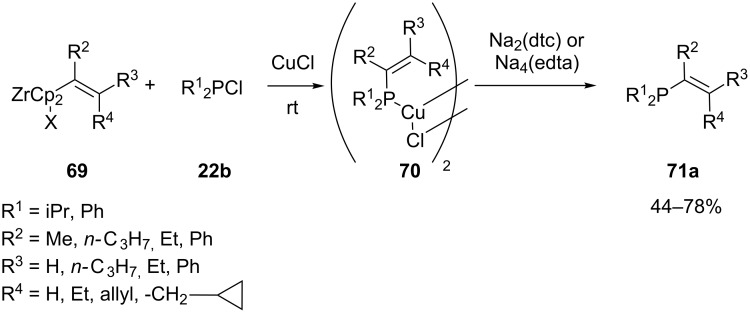
Preparation of alkenylphosphines **71a** from alkenylzirconocenes **69** (dtc = *N,N*-diethyldithiocarbamate, edta = ethylenediaminetetraacetate).

Alkenylphosphines were also synthesized by reacting alkenylzirconocenes **69** with a chlorophosphine **22b**. Alkenylzirconocene compounds **69** displaying different substitution patterns were used, giving access to a variety of alkenylphosphines **71a** via this method. If a more sterically hindered substrate ((α-substituted alkenyl)zirconocene) or reagent (iPr_2_PCl) is used, a transmetallation of Zr(IV) to Cu(I) is necessary for the reaction in order to proceed (Scheme 18). An intermediate phosphorus-copper complex **70** is formed. The phosphines **71a** were liberated by treatment with Na_2_(dtc) or Na_4_(edta) [145].

#### Nucleophilic substitution with metallated organophosphines

The method is based on the reaction of phosphorus nucleophiles, derived from secondary phosphines or phosphine–borane complexes, and carbon electrophiles. Nucleophilic substitution with metallated organophosphines is less frequently used for the synthesis of vinylphosphines [42,146] due to possible isomerization to phospha-alkenes under basic conditions [147]. The method is mainly applied for the synthesis of arylphosphines. However, the nucleophilic reagents are incompatible with functional groups susceptible to nucleophilic attack. These sensitive groups have to be protected first to avoid undesired reactions. Despite these limitations this approach is still generally used for the synthesis of simple phosphines [137–138,148–149].

**Scheme 19:**
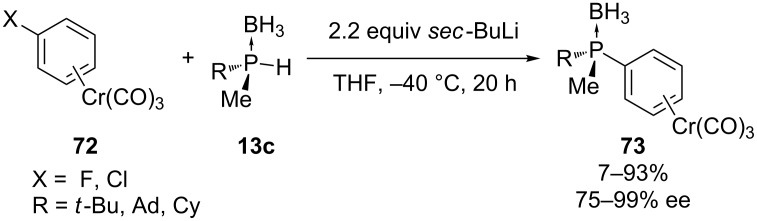
S_N_Ar with *P*-chiral alkylmethylphosphine boranes **13c**.

The group of Imamoto reported the S_N_Ar reaction of *P*-chiral secondary phosphine boranes **13c** with halobenzenechromium complexes **72** in the presence of *sec*-butyllithium [150]. The stereochemistry at the phosphorus atom was retained during the substitution when it was performed in THF at low temperature (Scheme 19). When fluorobenzenechromium complex **72** was used as a substrate, the yields of **73** were high (81–93%), in contrast to the reaction with chloro- and bromobenzenechromium complexes. The former reacted in low yield (7%), the latter did not react. The highly electronegative fluorine atom is needed for the S_N_Ar reaction to take place, even though the arenechromium complexes are already very electron-deficient aromatic compounds.

**Scheme 20:**
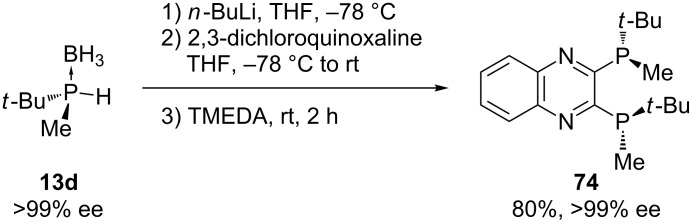
Synthesis of QuinoxP **74** (TMEDA = tetramethylethylenediamine).

The same group also developed a *P*-chiral ligand, QuinoxP **74**, via deprotonation of chiral secondary phosphine borane **13d** with *n*-butyllithium and subsequent nucleophilic substitution with 2,3-dichloroquinoxaline at low temperature (Scheme 20) [151]. After removal of the boranato group, the ligand was obtained in a good yield (80%).

#### Catalytic C(sp^2^)–P bond formation

The transition metal typically used for catalytic C–P bond formation is palladium [152] and, in some cases, nickel or copper. The phosphinating agents may comprise primary and secondary phosphines, silylphosphines [153] or phosphine–borane complexes.

The vinylic coupling partner mostly consists of alkenylhalides or alkenyltriflates. Vinyl triflates are used more since they can easily be derived from the corresponding ketone and they are more reactive then the vinyl chloride or bromide during the oxidative addition. More recently also vinyl tosylates and enol phosphates have proven to be suitable reagents.

The catalytic arylic C–P cross-coupling reaction can be a greener approach towards the widely used arylphosphines that are inaccessible by hydrophosphination. Recent advances in this area concern the synthesis of *P-*stereogenic phosphines through a dynamic kinetic resolution of racemic secondary phosphines in a metal-catalyzed P–H/aryl halide coupling.

#### C(sp^2^)–P bond formation of vinylphosphines

**Palladium:** Beletskaya and co-workers have described the synthesis of secondary and tertiary vinylphosphines by means of palladium catalyzed cross-coupling of vinylhalides and (silyl)phosphines [154–156]. Table 6 shows the protocols (A or B) generally used [157]. The vinylhalide substrates **75a** were cross-coupled with diphenylphosphine or diphenyltrimethylsilylphosphine. When diphenylphosphine was used, triethylamine was added for the basic activation of the phosphinating agent. All the tested substrates **75a** contained an alkoxy or amino group and depending on their position relative to the halogen, it was necessary to adjust the reaction temperature. The substrates bearing the halogen in the α-position to the alkoxy or amino group proved to be more reactive. With the halogen in β-position the substrate was less activated and the temperature had to be raised. Method B gave lower yields and longer reaction times were required to compensate for the use of the less reactive diphenyltrimethylsilylphosphine.

**Table 6:** Pd-catalyzed cross-coupling reactions of diphenylphosphine with alkenylhalides **75a**.

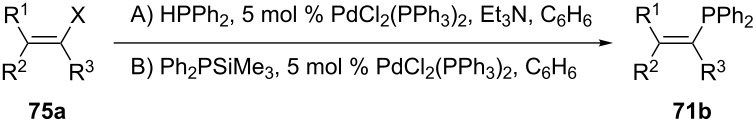								

Entry	R^1^	R^2^	R^3^	X	Method	Temp (°C)	Time (h)	Yield of **71b** (%)

1	H	H	OEt	Br	A B	20 20	1 1.5	97 92
2	Me	Me	NEt_2_	Cl	A B	20 20	6 12	84 80
3	H	OBu	Br	Br	A B	120 120	36 40	94 90
4	Ph	*N*-morpholine	H	Br	A B	70 70	24 50	92 60
5	Ph	*N*-piperidine	H	Br	A B	70 70	24 45	90 55

**Scheme 21:**
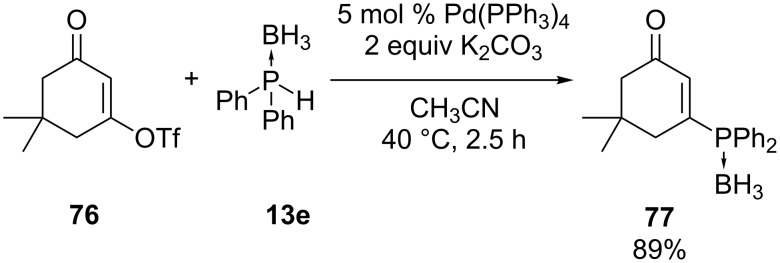
Pd-Mediated couplings of a vinyl triflate **76** with diphenylphosphine borane **13e**.

Lipshutz et al. used a Pd(0) catalyst to synthesize triarylphosphine boranes by coupling secondary diphenylphosphine borane **13e** with aryl nonaflates or triflates [158]. The article included one example with vinyl triflate **76** as a substrate (Scheme 21). The vinyl electrophile **76** was activated by the presence of the carbonyl group so the reaction also took place without a palladium catalyst albeit in lower yield (60%) and with formation of byproducts.

Julienne et al. have reported the coupling of secondary phosphine boranes with unactivated vinyl triflates (Table 7 and Table 8) [159]. Cyclic and acyclic vinyl triflates (**78** and **80a**) were reacted with diaryl-, dialkyl- and alkylarylphosphine–borane complexes, **13f** and **13g** respectively. The reactions were performed with a palladium catalyst in the presence of a weak base. Sometimes microwave irradiation was used to shorten the reaction time.

**Table 7:** Palladium-catalyzed C–P coupling between acyclic vinyl triflates and phosphine boranes (dppp = 1,3-bis(diphenylphosphino)propane).

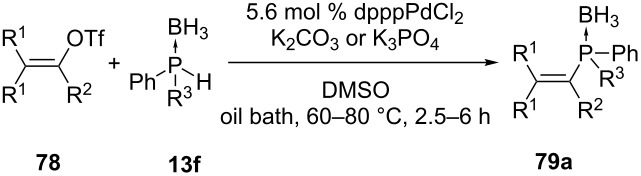				

Entry	R^1^	R^2^	R^3^	Yield of **79a** (%)

1	H	*t-*Bu	Ph	71
2	H	*t-*Bu	Me	72
3	Ph	Me	Ph	82
4	Ph	Me	Me	87

**Table 8:** Palladium-catalyzed C–P coupling between cyclic vinyl triflates and phosphine boranes (dppp = 1,3-bis(diphenylphosphino)propane).

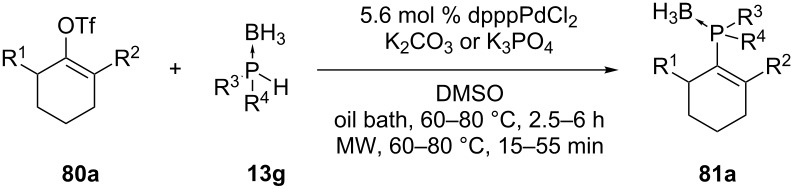						

Entry	R^1^	R^2^	R^3^	R^4^	Heating	Yield of **81a** (%)

1	H	H	Ph	Ph	Oil bath	68
2	H	H	Ph	Ph	MWI	71
3	H	H	Me	Ph	Oil bath	71
4	H	H	*t-*Bu	Ph	Oil bath	70
5	H	H	*t-*Bu	Ph	MWI	77
6	H	H	Et	Et	Oil bath	50
7	H	H	Cy	Cy	MWI	67
8	Me	H	Ph	Ph	Oil bath	70
9	H	Me	Ph	Ph	Oil bath	65

**Figure 1:**
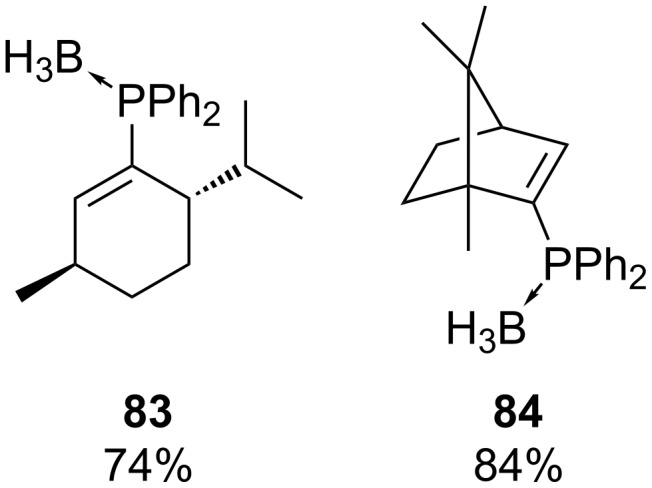
Menthone (**83**) and camphor (**84**) derived chiral phosphines.

Gilbertson et al. have converted a series of vinyl triflates **80b** into the corresponding vinyl phosphine boranes **81b** through palladium catalysis with HPPh_2_ (Table 9) [160]. The reaction proceeded under mild conditions (40 °C). These vinyltriflates **80b** were obtained from the corresponding ketone **82** opening access to a range of other structures. The chiral phosphines **83** and **84** were prepared from the natural products menthone and camphor in the same manner (Figure 1). All products were converted to the corresponding borane complex to facilitate further handling. However, when the same conditions were applied with diphenylphosphine borane and cyclohexenyltriflate no reaction was observed. A similar methodology has been applied for the synthesis of several ligands [161–163].

**Table 9:** Palladium-catalyzed synthesis of vinylphosphines **81b** from ketones **82** (dppb = 1,4-bis(diphenylphosphino)butane).

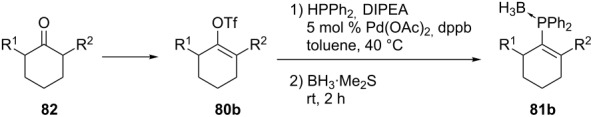			

Entry	R^1^	R^2^	Yield of **81b** (%)

1	H	H	96
2	Me	H	89
3	H	Me	89
4	*t-*Bu	H	88

**Scheme 22:**
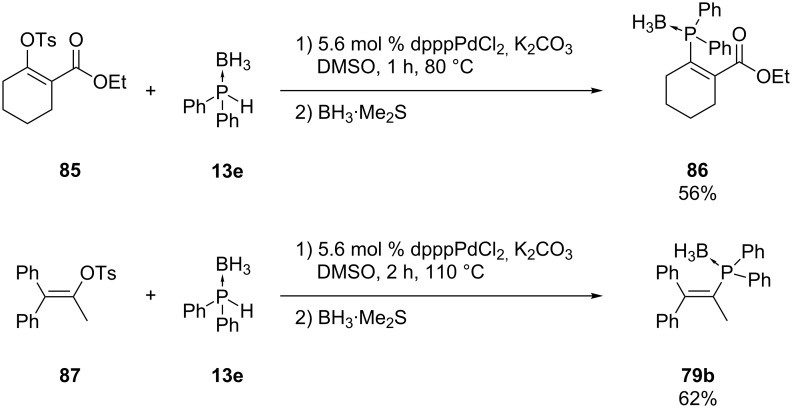
Palladium-catalyzed cross-coupling reaction of vinyl tosylates **85** and **87** with diphenylphosphine borane **13e** (dppp = 1,3-bis(diphenylphosphino)propane).

Julienne et al. succeeded in coupling vinyl tosylates **85** and **87** with diphenylphosphine borane **13e** despite the fact that alkenyl tosylates are poor reagents for cross-coupling [164]. The products **86** and **79b** were formed in the presence of a palladium catalyst. The reaction proceeded at lower temperature when the vinyl tosylate was substituted with an electron-withdrawing group like in **85** (Scheme 22).

**Scheme 23:**
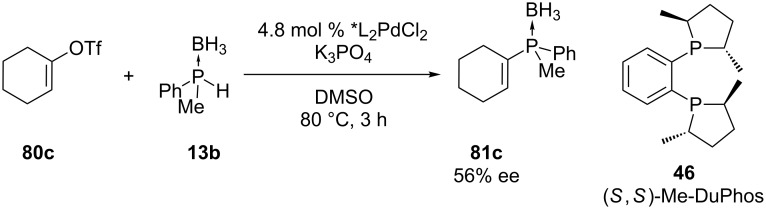
Attempt for the enantioselective palladium-catalyzed C–P cross-coupling reaction between an alkenyltriflate **80c** and a phosphine borane **13b**.

The group of Gaumont has also reported their preliminary results for the enantioselective palladium-catalyzed C–P cross-coupling reaction between an achiral vinyl triflate **80c** and a racemic secondary phosphine–borane complex **13b** (Scheme 23) [165]. Chiral phosphines with a *C*-stereogenic center have been studied but this was the first attempt for the asymmetric synthesis of a *P-*stereogenic compound. After evaluating several conditions the best catalyst was (*S*,*S*)-Me-DuPhos (**46**). An enantioenriched alkenylphosphine **81c** was formed. The highest enantiomeric excess measured by chiral HPLC was 56%. No reaction was observed without the palladium catalyst [165].

**Scheme 24:**
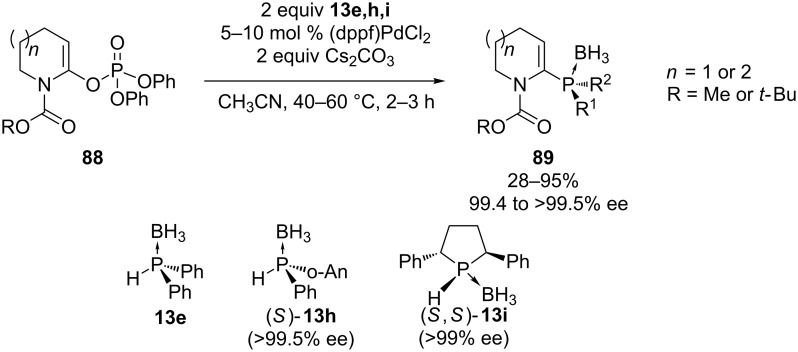
Enol phosphates **88** as vinylic coupling partners in the palladium-catalyzed C–P cross-coupling reaction (dppf = 1,1'-bis(diphenylphosphino)ferrocene).

Gillaizeau and co-workers have demonstrated the use of α-amido enol phosphates **88** as vinylic coupling partners in the palladium-catalyzed C–P cross-coupling reaction (Scheme 24) [166]. The enol phosphates **88** were prepared from the corresponding amides. The phosphane function was introduced in the α-position of the nitrogen. Several chiral and achiral secondary phosphine borane complexes **13** were used. The coupling was achieved under mild conditions. Most reactions gave **89** in low to good yields but in some cases the product could not be isolated, probably due to instability of the product. During the coupling reaction with **13h** partial inversion of the phosphorus atom occurred, resulting in racemization.

**Scheme 25:**
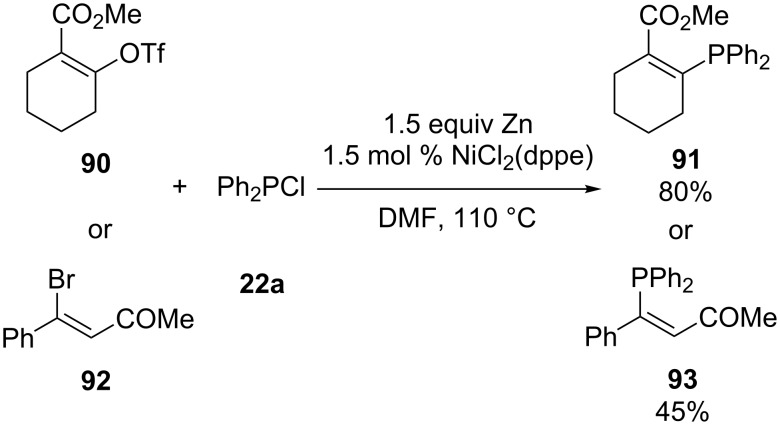
Nickel-catalyzed cross-coupling in the presence of zinc (dppe = 1,2-bis(diphenylphosphino)ethane).

**Nickel:** Most research has focused on the use of a palladium catalyst to perform the C–P cross-coupling between secondary phosphines and vinylic electrophiles. A few reports are available concerning the nickel-catalyzed cross-coupling. Ager and Laneman have prepared phosphines **91** and **93** from vinyl triflate **90** and vinyl bromide **92**, respectively, under similar conditions (Scheme 25) [53]. The reaction was catalyzed by NiCl_2_(dppe) in the presence of zinc. The role of zinc was to reduce Ni(II) to Ni(0) and to form Ph_2_PZnCl for the transmetallation step.

Kazankova and co-workers have explored the catalysts (Ph_3_P)_2_NiCl_2_ and Ni(acac)_2_ for the coupling of several vinyl bromides **75b** and chlorides with **25d** (Table 10). These reactions proceeded without the addition of zinc [167].

**Table 10:** Alternative nickel-catalysed cross-coupling without zinc (acac = acetylacetone).

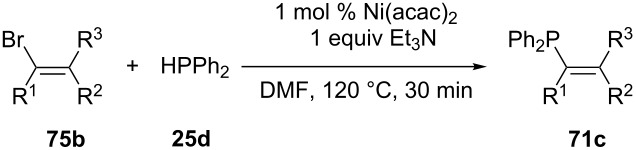				

Entry	R^1^	R^2^	R^3^	Yield of **71c** (%)

1	H	OEt	H	90
2	Me	H	Me	90
3	TMS	H	H	93
4	TES	H	H	96
5	H	Ph	H	90

**Scheme 26:**
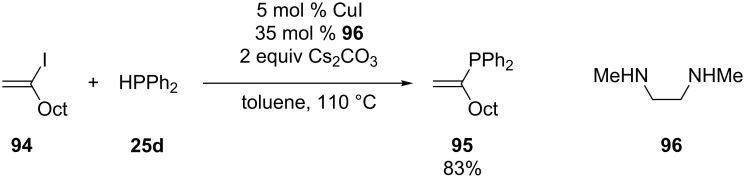
Copper-catalyzed coupling of secondary phosphines with vinyl halide **94**.

**Copper:** The group of Buchwald has reported one example of a copper catalyst to accomplish the phosphination of the vinyl halide **94** (Scheme 26) [168]. The protocol uses CuI as catalyst in combination with *N*,*N*’-dimethylethylenediamine (**96**) as ligand and a weak base Cs_2_CO_3_.The desired phosphine **95** is isolated in good yield.

#### C(sp^2^)–P bond formation of arylphosphines

The C–P bond formation of aryl phosphines is typically catalyzed by palladium, nickel and less frequently copper. The phosphorus coupling partners used are primary, secondary and tertiary phosphines, secondary phosphine–borane complexes, silyl- and stannylphosphines and phosphine chlorides. These phosphinating agents are coupled with aryl halides and triflates. Several general protocols are available.

**Scheme 27:**
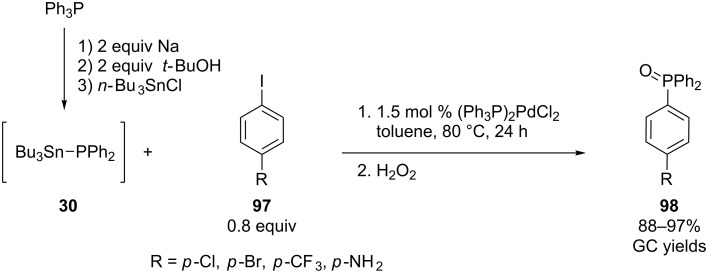
Palladium-catalyzed cross-coupling of aryl iodides **97** with organoheteroatom stannanes **30**.

**Palladium:** In 1987, Tunney and Stille reported on the palladium-catalyzed synthesis of several aryldiphenylphosphines by cross-coupling aryl halides with (trimethylsilyl)diphenylphosphine or (trimethylstannyl)diphenylphosphine [169]. No base is required for this method. Trimethylsilyl compounds are preferred over tristannyl derivatives since they are less toxic. However, in recent years the group of Rossi has reported a one-pot procedure for the palladium-catalyzed coupling of aryl iodides **97** with in situ generated Ph_2_SnBu_3_ (**30**, Scheme 27) [170]. When naphthyl triflate was used as a substrate, CuI was added as a co-catalyst [171].

**Scheme 28:**
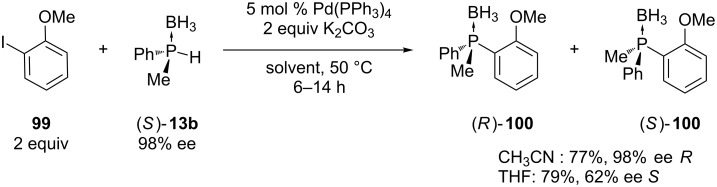
Synthesis of optically active phosphine boranes **100** by cross-coupling with a chiral phosphine borane **13b**.

Imamoto et al. have developed a method for the palladium-catalyzed C–P bond formation using secondary phosphine boranes [41]. The authors also discovered how the choice of the solvent influences the stereochemistry of **100**. When the coupling between aryl iodide **99** and asymmetric secondary phosphine borane **13b** was performed in acetonitrile or DMF, the stereochemistry at the phosphorus atom was almost completely retained while the reaction performed in THF or toluene resulted mainly in inversion (Scheme 28) [172–173]. The stereochemistry also depended on the base used. The presence of K_2_CO_3_ or KOAc favored a good stereoselectivity in contrast to K_3_PO_4_ or DBU. Sodium hydride or Ag_2_CO_3_ promoted retention of configuration. The mechanism of the reaction was studied by Gaumont et al. through isolation of the reactive intermediate [174]. Lipshutz et al. reported the palladium-catalyzed phosphination of aryl triflates and nonaflates instead of aryl iodides with phosphine boranes [158]. The first examination towards an enantioselective C–P cross-coupling starting from racemic secondary phosphine boranes was performed by Gaumont and Pican [175]. The highest enantiomeric excess obtained was 45%. The same group has shown that imidazolium based ionic liquids can be used as a medium to perform the C–P cross-coupling reactions. This method allows an easy separation of the product from the catalyst and the recycling of the palladium catalyst [176].

**Scheme 29:**
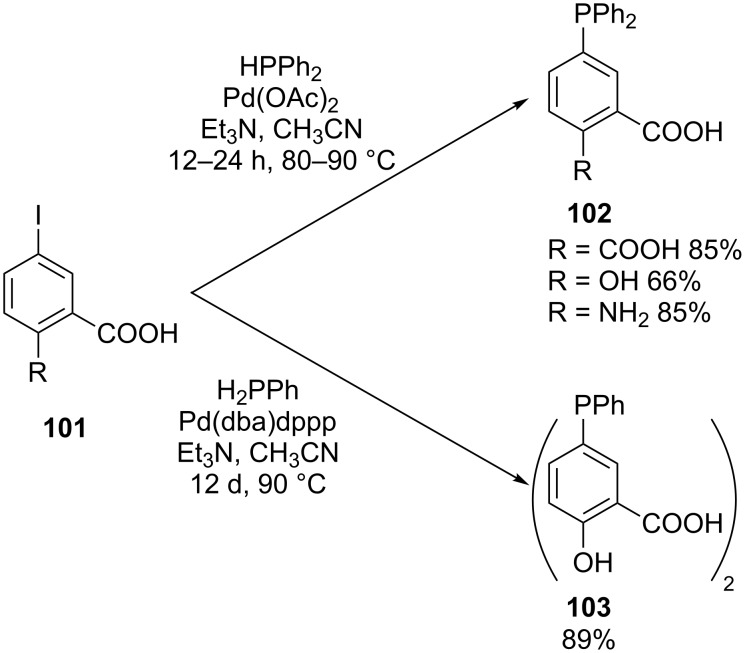
Palladium-catalyzed P–C cross-coupling reactions between primary or secondary phosphines and functional aryliodides **101** (dba = dibenzylideneacetone, dppp = 1,3-bis(diphenylphosphino)propane).

Stelzer and co-workers have developed a general method for the coupling of primary or secondary phosphines instead of their silyl derivatives or borane complexes with functional aryliodides **101** [177–179]. It should be noted, however, that the reactions were again limited to (di)phenylphosphine (Scheme 29). The protocols use palladium as a catalyst in the presence of tertiary amines as base. A variety of hydrophilic phosphines (**102**, **103**) was synthesized. Since no protective groups were introduced, the method proves to be compatible with several functionalities. This methodology or in a slightly modified form has been used by several authors for the phosphination of a large variety of compounds [180–188]. Microwave-assisted procedures have also been developed [189–191].

Kwong et al. implemented a palladium-catalyzed phosphination of aryl bromides and triflates **104** with triarylphosphines **105a** as phosphinating agents. This aryl–aryl exchange reaction was compatible with several functional groups such as ketones, aldehydes, esters, nitriles, ethers (Table 11) [192–195]. Products **106a** were isolated in only moderate yields. Several *P*,*N*-biaryl ligands were prepared from the corresponding triflate under similar conditions [196–197]. The reaction also proceeded under solvent-free conditions with slightly higher yields [198]. A heterogeneous Pd/C catalyst has been applied as well [199–200].

**Table 11:** The phosphination of aryl bromides **104** with tertiary arylphosphines **105a**.

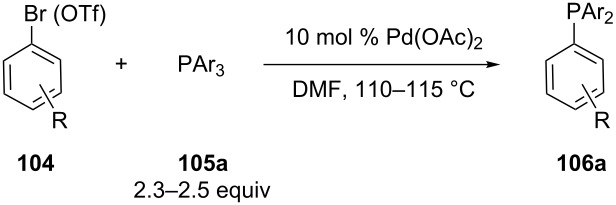			

Entry	R	Ar	Yield of **106a** (%)

1	-CHO	Ph	32
2	-C(O)Me	Ph	40
3	-CO_2_Me	Ph	30
4	-CN	Ph	36
5	-OMe	Ph	27
6	-C(O)Me	*p*-Tol	39
7	-C(O)Me	3,5-Me_2_-C_6_H_3_	34
8	-C(O)Me	*p*-MeO-C_6_H_4_	33

**Scheme 30:**
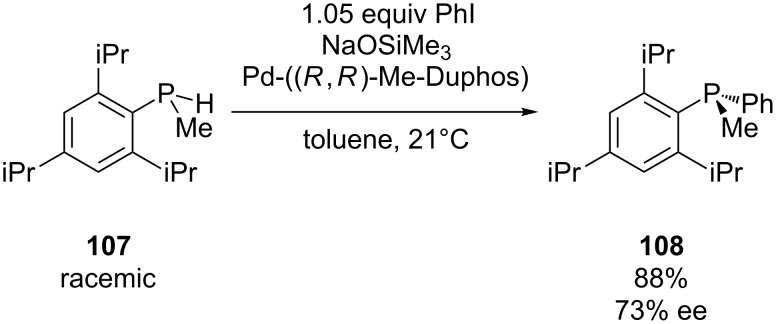
Enantioselective synthesis of a *P-*chirogenic phosphine **108**.

The group of Glueck has reported the first asymmetric palladium-catalyzed C–P bond formation for the synthesis of *P-*stereogenic phosphines by adding a catalytic amount of a chiral auxiliary. The enantioenriched phosphine **108** was obtained through coupling of racemic bulky secondary phosphine **107** with PhI in the presence of the base NaOSiMe_3_ and the Pd-catalyst (Scheme 30) [201]. In the following years, the scope and mechanism were elaborated [202–204]. In accordance with the mechanism given in Scheme 10, it was concluded that the major enantiomer of the product **108** was derived from the major diastereomer of the Pd-phosphido intermediate. Korff and Helmchen have prepared several triarylphosphines with this methodology. However, a modified catalyst system [Pd(Et-FerroTANE)] containing a ferrocene-based ligand was used [205]. This catalyst had the advantage that it was easily prepared in situ while the unstable catalyst used by Glueck et al., required storage at −25 °C in the dark.

**Scheme 31:**
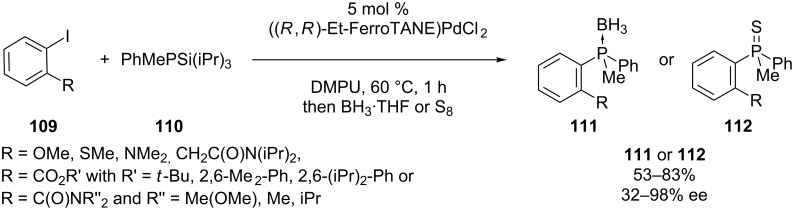
Enantioselective arylation of silylphosphine **110** ((*R,R*)-Et-FerroTANE = 1,1'-bis((*2R*,*4R*)-2,4-diethylphosphotano)ferrocene).

The protocol of Tunney and Stille starting from silylphosphines has been modified by Chan, Bergman and Toste to be enantioselective by using a [Pd(Et-FerroTANE)] catalyst. *P-*stereogenic phosphine boranes **111** and **112** were synthesized by arylation of racemic silylphosphines **110** under dynamic kinetic control (Scheme 31). The best enantiomeric excess was obtained when an *ortho*-amide substituent was present in the substrate **109** [206].

**Scheme 32:**
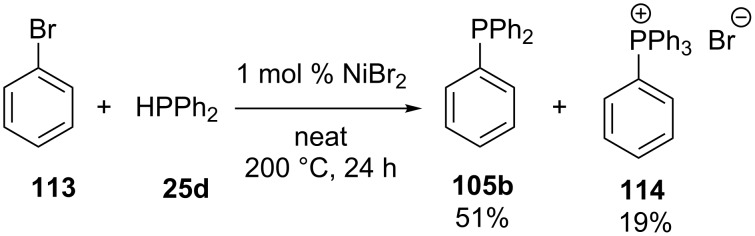
Nickel-catalyzed arylation of diphenylphosphine **25d**.

**Nickel:** Cristau et al. were the first which achieved the nickel-catalyzed arylation of diphenylphosphine [207]. Upon reaction of bromobenzene (**113**) with **25d** in the presence of NiBr_2_ a mixture of triphenylphosphine **105b** and tetraphenylphosphonium bromide salt **114** was obtained (Scheme 32).

**Scheme 33:**
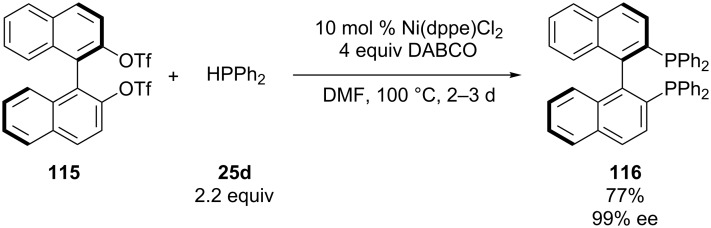
Nickel-catalyzed synthesis of (*R*)-BINAP **116** (dppe = 1,2-bis(diphenylphosphino)ethane, DABCO = 1,4-diazabicyclo[2.2.2]octane).

The first conversion of an aryltriflate to an arylphosphine using diphenylphosphine was reported by Cai et al. (Scheme 33) [208–209]. The method was developed for the synthesis of chiral (*R*)-BINAP **116**; a successful chiral ligand. Nickel was chosen as catalyst instead of palladium to minimize catalyst poisoning by binding of the metal with the phosphines present. After optimization, the desired chiral BINAP **116** was obtained in 77% yield. This protocol has been adopted by other research groups for the synthesis of a range of phosphines [138,210–216]. Analogous palladium-catalyzed reactions coupling aryl triflates with diphenylphosphine have been reported [217–218].

Laneman et al. later developed a modified version of Cai’s method and synthesized several tertiary phosphines **118** via the cross-coupling of aryl triflates and halides **117** with chlorodiphenylphosphine (**22a**) instead of diphenylphosphine (Table 12) [53]. The reaction was catalyzed by NiCl_2_(dppe) in the presence of zinc. A hydrodehalogenation side reaction resulted in lower yields of aryl halide substrates compared to aryl triflates.

**Table 12:** Preparation of tertiary phosphines **118** via nickel-catalyzed cross-coupling (dppe = 1,2-bis(diphenylphosphino)ethane).

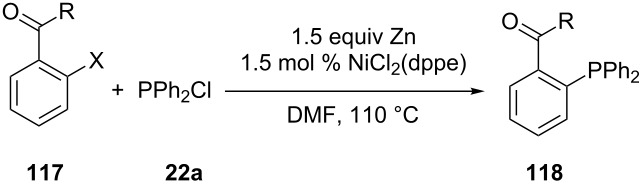			

Entry	R	X	Yield of **118** (%)

1	OMe	OTf	84
2	OMe	Br	46
3	NHBn	OTf	67
4	(*S*)-NHCHMePh	Br	46

**Scheme 34:**
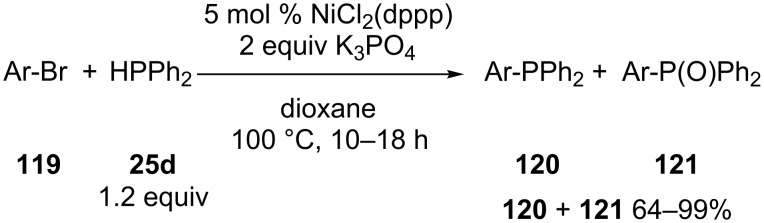
Nickel-catalyzed cross-coupling between aryl bromides **119** and diphenylphosphine (**25d**) (dppp = 1,3-bis(diphenylphosphino)propane).

Zhao and co-workers disclosed a method for the cross coupling of various aryl bromides **119** with diphenylphosphine (**25d**) in the absence of external reductants and supporting ligands [219]. The reaction gave mixtures of phosphines **120** and phosphine oxides **121** (Scheme 34). Several functional groups (ester, ether, ketone and cyano groups) remained intact under the conditions. The reaction was also performed with diphenylphosphine–borane complex but this resulted in only small amounts of products due to decomposition of the phosphinating reagent at 100 °C.

**Scheme 35:**
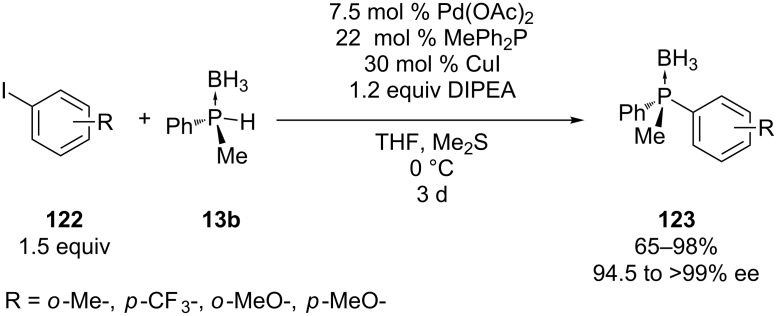
Stereocontrolled Pd(0)−Cu(I) cocatalyzed aromatic phosphorylation.

**Copper:** Copper was first used as a co-catalyst in palladium-catalyzed phosphorylation reactions, Livinghouse et al. demonstrated that the aromatic phosphorylation proceeded even at low temperatures of ≤0 °C when copper was added [220]. The method also allows for the stereocontrolled Pd(0)−Cu(I) co-catalyzed coupling of enantiopure secondary phosphine borane **13b** with aryl iodides **122** (Scheme 35) [221].

In 2003, copper-catalyzed palladium free phosphorylation methods were developed by Venkataraman and Van Allen [222] and Buchwald et al. [168]. Both methodologies use catalytic amounts of copper(I) salts in the presence of K_2_CO_3_ or Cs_2_CO_3_ as a base. Buchwald et al. also added *N*,*N*’-dimethylethylenediamine **96** as a ligand to enhance the efficiency of the coupling. A secondary phosphine **25e** was coupled with a variety of aryl halides **124** with electron-withdrawing or -donating substituents. The method tolerated the presence of functional groups such as esters or amines (Table 13). This approach was also used for the synthesis of phosphinoxazolines [223].

**Table 13:** Copper-catalyzed synthesis of triarylphosphines **106b**.

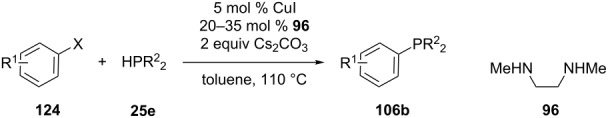				

Entry	R^1^	X	R^2^	Yield of **106b** (%)

1	2-MeO	I	Ph	91
2	2-NH_2_	I	Ph	86
3	4-CO_2_Me	Br	Ph	70
4	2-Ph	I	tol	79
5	4-NH_2_	I	Cy	72
6	4-CO_2_Et	I	Cy	85
7	4-CN	I	iBu	65

#### Hydrophosphination of alkynes

The addition of P–H to a triple bond is a highly desirable method when taking atom economy principles into account. Activated [224–225] or unactivated alkynes were investigated as substrates. Phosphines as well as silylphosphines [65–66,226–227] or phosphine–borane complexes can be used as phosphinating agents. The addition reaction has been initiated in several ways including base [228–233], radical (thermal radical [234] or AIBN radical [77–78,83,235–236]) or transition metal activation.

**Scheme 36:**
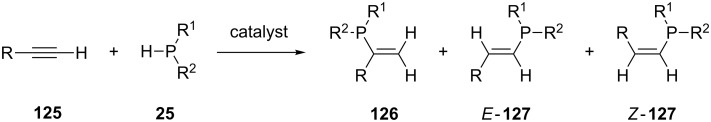
Preparation of alkenylphosphines by hydrophosphination of alkynes.

Depending on the regioselectivity of the procedure, the addition of P–H to the triple bond results in the formation of two regioisomers (Scheme 36). The product that results from the Markovnikov addition of P–H corresponds to the α-adduct **126** and the anti-Markovnikov addition results in the β-adduct **127**. The stereoselectivity of the reaction determines the formation of *E-* or *Z*-**127**.

Despite the great appeal of this method for the preparation of vinylphosphines it does not allow the syntheses of the widely used arylphosphines or alkenes bearing no hydrogen on the double bond. Additionally, due to the absence of small rings containing a triple bond, no cyclic alkenylphosphines are accessible. Until now, the protocols lack sufficient control over selectivity and mostly give mixtures. Most addition products (radical, base, metal) are anti-Markovnikov **127**, only a few palladium catalyzed reactions give the Markovnikov products **126**.

Several reviews on hydrophosphination of alkynes have been published [90–91,237]. Some recent developments will be discussed. In recent years research has mainly focused on metal-catalyzed hydrophosphinations.

#### Metal complex-catalyzed hydrophosphinations

Hydrophosphination catalysts are mainly based on transition metals. However, it has been shown that lanthanides and alkaline earth metals can offer a valid alternative.

**Scheme 37:**
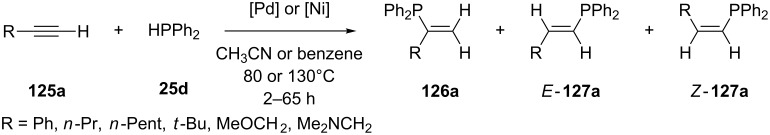
Palladium and nickel-catalyzed addition of P–H to alkynes **125a**.

Palladium and nickel complexes were used to catalyze the addition of the P–H bond to alkynes **125a** (Scheme 37). The regioselectivity was strongly dependent on the catalytic precursor. In the presence of palladium(0) and nickel(0) complexes the β-adduct **127a** was formed as the major product. By contrast palladium(II) and nickel(II) complexes mainly gave rise to the α-adduct **126a** [98,238]. The nickel based catalyst was more effective than the palladium so the reaction proceeded at lower temperature.

**Scheme 38:**
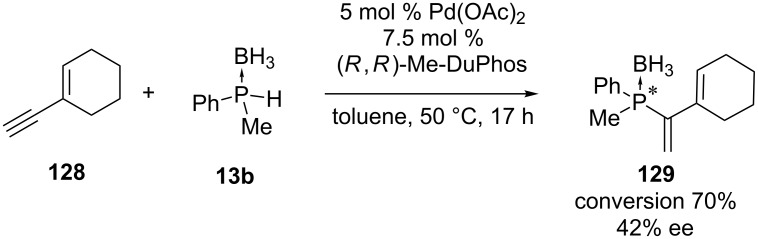
Palladium-catalyzed asymmetric hydrophosphination of an alkyne **128**.

Join et al. had the objective to enantioselectively create *P-*stereogenic vinylphosphine boranes [239]. To achieve this goal some asymmetric hydrophosphination reactions were performed using a palladium catalyst in combination with a chiral ligand. After optimizing the conditions, the addition of methylphenylphosphine borane (**13b**) to 1-ethynylcyclohexene (**128**) with the Pd-catalyst afforded tertiary phosphine borane **129** with a conversion of 70% and only 42% ee (Scheme 38).

Nagata et al. performed the palladium-catalyzed hydrophosphination of alkynes by using tetraphenyldiphospine (**130**) (Table 14) [240]. Since there is no P–H bond in this phosphinating agent, a bisphosphination was expected but a hydrophosphination took place. However, an excess (3–5 equiv) of alkyne was used. The reaction proceeded regioselectively and the α-adducts **126b** of several terminal alkynes **125b** were formed. Air-oxidation during work-up resulted in the formation of the corresponding phosphine oxides **131**. The products **131** were isolated in moderate yields with respect to the diphosphine **130** as limiting reagent. It was suggested that the alkynyl hydrogen acts as the hydrogen source for the hydrophosphination. This can also explain why the method was not applicable to internal alkynes. Silanes have also been added as the source for hydrogen [241].

**Table 14:** Pd-catalyzed hydrophosphination of alkynes **125b** using diphosphine **130**.

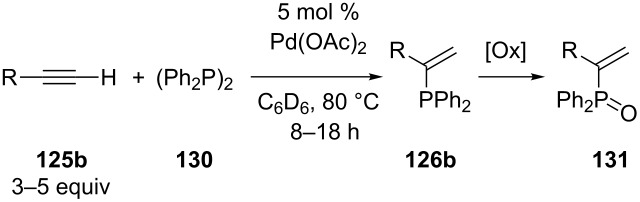		

Entry	R	Yield of **131** (%)

1	*n-*Hex	58
2	Ph	66
3	-(CH_2_)_3_CN	50
4	-(CH_2_)_3_Cl	75

**Scheme 39:**
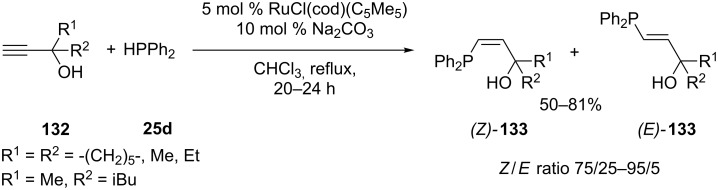
Ruthenium catalyzed hydrophosphination of propargyl alcohols **132** (cod = 1,5-cyclooctadiene).

Ruthenium complexes are the first catalysts reported for the direct hydrophosphination of propargyl alcohols [242]. Several catalytic systems were tested and the reaction with 5 mol % RuCl(cod)(C_5_Me_5_) in the presence of Na_2_CO_3_ provided the best results (Scheme 39). The reaction gave two stereoisomeric adducts (*Z)*-**133** and (*E)*-**133**. The hydrophosphination of **132** proceeded with excellent regioselectivity and good stereoselectivity as the *Z*-isomers, (*Z*)-**133**, were preferentially formed with *Z*/*E* ratios around 80/20. This method could not be performed on alkynes with an internal triple bond, only terminal alkynes were accessible.

**Scheme 40:**
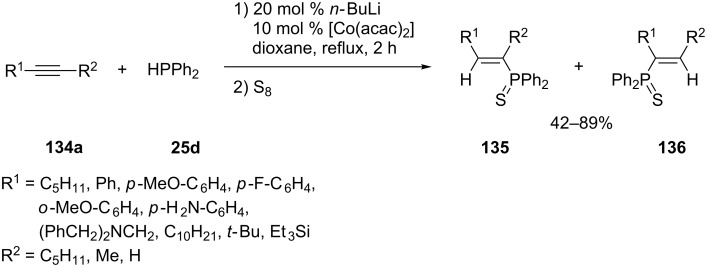
Cobalt-catalyzed hydrophosphination of alkynes **134a** (acac = acetylacetone).

A catalytic amount of Co(acac)_2_ in combination with butyllithium can mediate the hydrophosphination of internal alkynes [243]. Various alkynes **134a** were subjected to these conditions to provide the corresponding *syn*-adducts exclusively (Scheme 40). The regioselectivity is mostly influenced by steric hindrance. To avoid loss of product by oxidation, the adducts were isolated as their thiophosphine analogues **135** and **136**.

Hayashi and co-workers have reported a rhodium-catalyzed phosphination of alkynes **134b** using silylphosphines **137** as phosphinating agents (Table 15) [108]. The cationic rhodium catalyst was generated in situ by adding silver triflate to a chlororhodium complex. The silylgroup was not incorporated in the vinylphosphine product **138a** and methanol was added as a proton source for completing the reaction. The adducts **138a** were formed with good to high *syn*-selectivity.

**Table 15:** Rhodium-catalyzed hydrophosphination of alkynes **134b** with a silylphosphine **137** (cod = 1,5-cyclooctadiene).

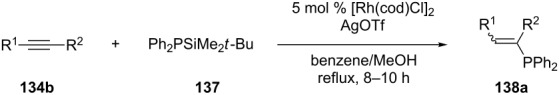				

Entry	R^1^	R^2^	Yield of **138a** (%)	*E*/*Z*

1	Ph	H	89	96/4
2	MeO-C_6_H_4_	H	53	92/8
3	*n-*C_5_H_11_	H	78	95/5
4	HOCH_2_	H	66	80/20
5	Ph	Me	68	92/8
6	Ph	*n-*Bu	72	95/5
7	*n-*C_5_H_11_	*n-*C_5_H_11_	67	>99/1
8	EtO_2_C	*n-*Bu	81	>99/1
9	EtO_2_C	Ph	76	80/20

Kondoh et al. demonstrated the P–H addition to 1-alkynylphosphines under copper catalysis (Table 16) [244]. Besides copper(I) iodide several other copper salts effectuated the reaction albeit in lower yields as did silver(I) iodide, palladium(II) chloride and platinum(II) chloride. Other transition metal catalysts such as gold(I) chloride, nickel(II) chloride and cobalt(II) chloride gave no reaction. In the presence of copper(I) iodide and cesium carbonate diphenylphospine (**25d**) added to the triple bond in an *anti*-fashion. A diverse set of alkynylphosphines **139** was subjected to the protocol proving the compatibility of the method with certain functional groups. The *Z*-adducts were formed exclusively and isolated as the phosphine sulfides **140** to prevent lower yields by oxidation to the corresponding oxides. The phosphines **141** were obtained by radical reduction of **140** with tris(trimethylsilyl)silane (TTMSS).

**Table 16:** Copper-catalyzed hydrophosphination of 1-alkynylphosphines **139**.

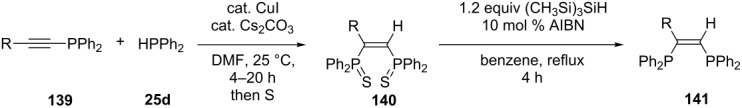			

Entry	R	Yield of **140** (%)	Yield of **141** (%)

1	*n-*Hex	88	87
2	iPr	84	
3	*t-*Bu	84	89
4	Ph	72	78
5	4-Ac-C_6_H_4_	87	63
6	3-pyridyl	62	44
7	EtOC(O)(CH_2_)_3_	79	
8	AcS(CH_2_)_9_	75	
9	PhCH(OH)	84	

**Scheme 41:**
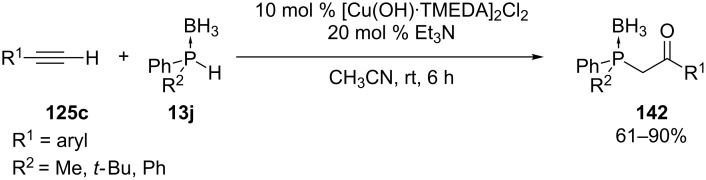
Tandem phosphorus–carbon bond formation–oxyfunctionalization of substituted phenylacetylenes **125c** (TMEDA = tetramethylethylenediamine).

However, when Kumaraswamy et al. explored the copper-catalyzed hydrophosphination on substituted phenylacetylenes **125c** further oxidation of the double bond led to the corresponding phenacyl tertiary phosphine boranes **142** in moderate to good yields (Scheme 41). The products **142** were obtained when the reactions were performed under inert atmosphere and in open air. Since the latter gave slightly better yields, it was argued that the dissolved air contributed to the product formation. A Cu(II)–TMEDA catalyzed tandem phosphorus–carbon bond formation–oxyfunctionalization was developed [245]. When methyl propiolate was subjected to the same reaction conditions only the β-adducts were isolated.

**Scheme 42:**
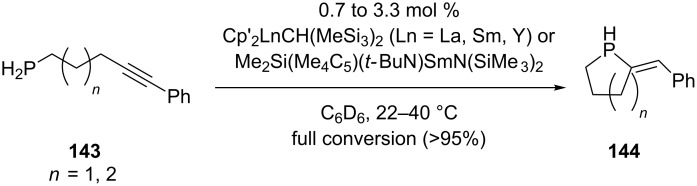
Organolanthanide-catalyzed intramolecular hydrophosphination/cyclization of phosphinoalkynes **143**.

The intramolecular hydrophosphination and cyclization of primary alkynyl phosphines **143** has been accomplished using organolanthanide precatalysts of the type Cp’_2_LnCH(SiMe_3_)_2_ (Cp’ = *η*^5^-C_5_Me_5_) and Me_2_Si(Me_4_C_5_)(*t-*BuN)SmN(SiMe_3_)_2_ [111–112]. The reaction succeeded also using homoleptic lanthanocenes of the form Ln[CH(SiMe_3_)_2_]_3_ (Ln = La, Nd, Sm, Y, Lu) or Ln[N(SiMe_3_)_2_]_3_ (Ln = La, Nd, Sm, Y) [246]. The reaction was performed in NMR tubes until full conversion to the phospholane **144** (*n* = 1) or phosphorinane **144** (*n* = 2) was obtained (Scheme 42). The reaction is regioselective as only one adduct was obtained. Several butadiene derivatives were synthesized by hydrophosphination of the triple bond in enynes in the presence of yttriumcomplexes [247].

**Scheme 43:**
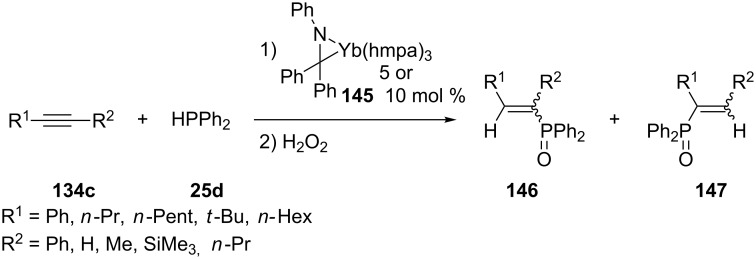
Hydrophosphination of alkynes **134c** catalyzed by ytterbium-imine complexes **145** (hmpa = hexamethylphosphoramide).

An ytterbium–imine complex **145** [Yb(*η*^2^-Ph_2_CNPh)(hmpa)_3_] has also been applied for the synthesis of alkenylphosphines [245,248–251]. The products were isolated as their corresponding phosphine oxides (**146** and **147**) after oxidative work-up (Scheme 43). The reaction proceeded under mild conditions (rt, 5 min to 4 h), except for the less reactive aliphatic internal alkynes (80 °C, 6 h). The regio- and stereoselectivity was mainly affected by the nature of the substrate and not so much by the reaction conditions. An active ytterbium phosphide species is generated in situ and therefore the imine complex could be categorized as a basic catalyst.

**Scheme 44:**
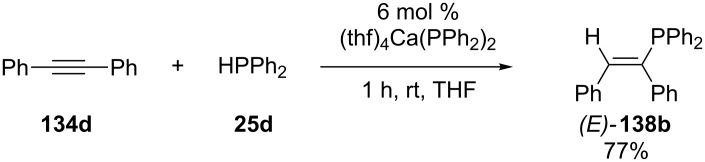
Calcium-mediated hydrophosphanylation of alkyne **134d**.

The only catalysts based on heavy alkaline earth metals for the hydrophosphination of alkynes are derived from calcium [123,252–253]. A similar behavior of calcium(II) and ytterbium(II) compounds seems possible as the oxidation state of Yb(II) does not change during the ytterbium(II)-catalyzed hydrophosphination of alkynes. The reaction of alkyne **134d** in the presence of the calcium catalyst resulted in diphenyl-vinylphosphine **138b** in good yield (Scheme 44). A set of butadiynes was reacted in a similar way [254]. Mixtures of butadienyldiphosphine isomers were obtained depending on the bulkiness of the end groups at the butadiyne moieties.

#### Other hydrophosphinations

A relatively recent example for the thermal activated hydrophosphination was from Mimeau and Gaumont and described the use of a microwave reactor [254]. This reaction is performed with secondary phosphine–borane complexes **13j** and terminal alkynes **125d**. Mimeau and Gaumont demonstrated that the regioselectivity of the hydrophosphination reaction can be controlled by adjusting the activation method. Thermal activation with the microwave reactor gave the β-adducts **148** (anti-Markovnikov addition) (Table 17). In the same article the α-adducts **149** (Markovnikov addition) were formed by using a palladium catalyst (Table 18). In both cases the regioselectivity was excellent, the stereochemistry in the case of the β-adduct **148** favoured the *Z*-product. The conditions are compatible with aliphatic and oxygen-functionalized alkynes.

**Table 17:** Hydrophosphination reactions of terminal alkynes **125d** with phosphine boranes **13j** under microwave conditions.

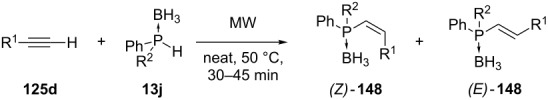				

Entry	R^1^	R^2^	Yield of **148** (%)	*Z*/*E* ratio

1	*n*-Hex	Ph	76	>95/5
2	Ph	Ph	0	
3	(CH_2_)_2_OH	Ph	49	>95/5
4	CH_2_OCH_3_	Ph	33	>95/5
5	*n*-Hex	Me	82	80/20
6	*n*-Hex	*t-*Bu	49	70/30

**Table 18:** Hydrophosphination reactions of terminal alkynes **125e** with phosphine boranes **13f** using a Pd catalyst (dba = dibenzylideneacetone, dppp = 1,3-bis(diphenylphosphino)propane).

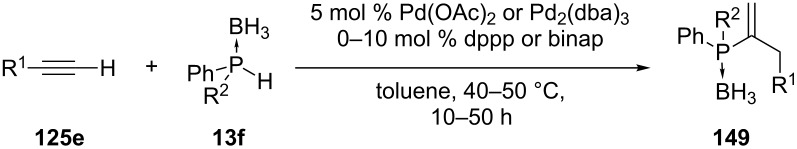			

Entry	R^1^	R^2^	Yield of **149** (%)

1	*n*-Hex	Ph	84
2	Ph	Ph	49
3	-(CH_2_)_2_OH	Ph	71
4	-CH_2_OCH_3_	Ph	73
5	Cy	Ph	60
6	*n*-Hex	Me	85
7	Ph	Me	53

Busacca et al. have described the hydrophosphination of internal alkynes with phosphine–borane complexes under basic conditions [255–256]. Several diaryl- and alkylarylalkynes **134e** were reacted with a variety of phosphine boranes **25f**, some examples are shown in Table 19. Mixtures of *E* and *Z*-isomers of **150** were formed, with the *E*-isomer as the major product.

**Table 19:** Hydrophosphination of alkynes **134e** with phosphine–borane complexes **25f** (DMAc = dimethylacetamide).

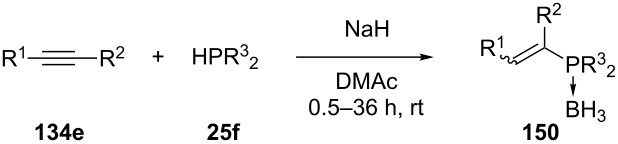					

Entry	R^1^	R^2^	R^3^	Yield of **150** (%)	*E/Z* ratio

1	Ph	Me	Cy	85	>20/1
2	Ph	Ph	*t-*Bu	88	>20/1
3	Ph	Me	*p*-(iPrO)-C_6_H_4_	78	>20/1
4	Ph	Ph	iBu	79	4/1
5	*p*-CF_3_-C_6_H_4_	*p*-CF_3_-C_6_H_4_	Ph	98	>20/1
6	*o*-Tol	*o*-Tol	Cy	99	>20/1

### Preparation of alkynylphosphines via formation of a C(sp)–P bond

An extensive review concerning the stoichiometric and catalytic synthesis of alkynylphosphines and their borane complexes has been published in 2012 by Gaumont et al. [257].

#### Reaction of organometallic reagents with halophosphines

Alkynylphosphines are commonly synthesized by the nucleophilic displacement of the halogen at the phosphorus atom of a halophosphine with a metal acetylide. Grignard [258–259] and organolithium [244,260–262] reagents have frequently been used since many years. The main disadvantage is the incompatability of lithium and magnesium reagents with alkynylphosphines having labile functional groups susceptible to nucleophilic attack.

This approach is mainly used for the synthesis of tertiary phosphines. It is difficult to synthesize secondary alkynylphosphines since they easily convert into their phosphaallene tautomer. They can only be obtained when they have sterically hindering substituents [263–264].

**Scheme 45:**
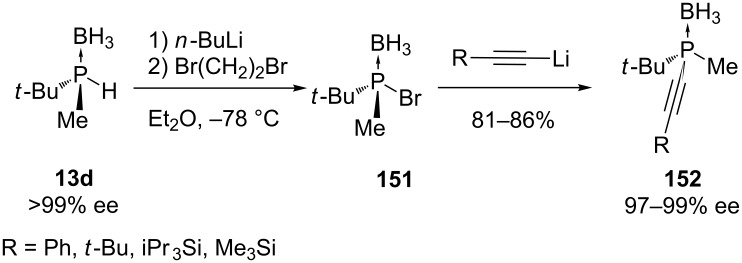
Formation and substitution of bromophosphine borane **151**.

The asymmetric synthesis of alkynylphosphines also suffers from limited availability of unsymmetrical halophosphines and their weak configurational stability. Stereospecific substitution at chiral phosphorus atoms by alkynyl nucleophiles has been reported by Imamoto et al. (Scheme 45) [265]. Firstly, a bromo(*tert-*butyl)methylphosphanyl borane **151** was formed in situ by treating the enantiomerically pure (*S*)-(*tert*-butyl)methylphosphine borane **13d** with *n-*BuLi and 1,2-dibromoethane. An alkynyl lithium reagent was directly added to intermediate **151**. The expected substitution products **152** were obtained in high yield and almost exclusively with inversion of configuration, resulting in excellent stereospecificities.

#### Catalytic C(sp)–P bond formation

**Scheme 46:**
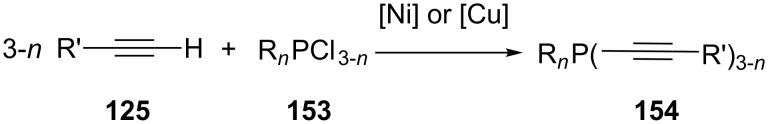
General scheme for a nickel or copper catalyzed cross-coupling reaction.

This type of carbon–phosphorus bond formation relies on the cross-coupling reaction in the presence of a catalyst. The cross-coupling reaction is in general performed between a terminal alkyne **125** and an electrophilic phosphorus reagent in the form of a halophoshine **153**, mostly chlorophosphine, in the presence of a catalyst such as nickel (Ni(acac)_2_) [244,266–267] or copper (CuI) [268–270] (Scheme 46). The nickel based catalyst was not suitable for the cross-coupling of alkynes containing a sensitive alkoxy or amino functional group. Therefore, another catalytic method was developed using copper(I) salts.

**Scheme 47:**
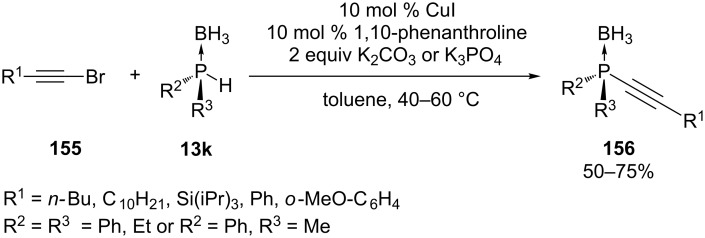
Copper-catalyzed synthesis of alkynylphosphines **156**.

Alkynylphosphines were synthesized through the use of a copper-catalyzed reaction between a secondary phosphine borane **13k** and various 1-bromoalkynes **155** in the presence of 1,10-phenanthroline as a ligand and K_2_CO_3_ or K_3_PO_4_ as a base (Scheme 47). This was the first method involving a nucleophilic phosphorus reagent in the synthesis of alkynylphosphines and was presented by the group of Gaumont [271–272]. The method was applicable for dialkyl, diaryl or alkylaryl phosphine boranes **13k** and required only mild conditions.

## Conclusion

The developments over the past years in the field were reviewed. The use of phosphines as ligands in metal complex catalysis has been a major driving force for the synthesis of functionalized phosphines. In recent years many catalytic procedures have emerged. In general these catalytic protocols proceed under milder conditions that tolerate the presence of functional groups. Gradually a broader variety of phosphines is accessible. Due to the growing importance of asymmetric catalysis, a lot of attention has been paid to the asymmetric synthesis of chiral phosphines. The challenge to find a general protocol that permits simple access to chiral phosphines, is still ongoing and further developments are required.
